# Longer procoagulant phospholipid-dependent clotting time, lower endogenous thrombin potential and higher tissue factor pathway inhibitor concentrations are associated with increased VTE occurrence in patients with newly diagnosed multiple myeloma: results of the prospective ROADMAP-MM-CAT study

**DOI:** 10.1038/s41408-018-0135-y

**Published:** 2018-11-07

**Authors:** Despina Fotiou, Theodoros N. Sergentanis, Loula Papageorgiou, Kimon Stamatelopoulos, Maria Gavriatopoulou, Efstathios Kastritis, Theodora Psaltopoulou, Stella Salta, Patrick Van Dreden, Rabiatou Sangare, Annette K. Larsen, Evangelos Terpos, Ismail Elalamy, Meletios A. Dimopoulos, Grigoris T. Gerotziafas

**Affiliations:** 1 Department of Clinical Therapeutics, National and Kapodistrian University of Athens, School of Medicine, Athens, Greece; 2 Sorbonne Universities, Faculty of Medicine, Cancer, Haemostasis and Angiogenesis Research Group, INSERM U938, Institut Universitaire de Cancérologie, Paris, France; 3 Service d’Hématologie Biologique Hôpital Tenon, Hôpitaux Universitaires de l’Est Parisien, Assistance Publique Hôpitaux de Paris, Paris, France; 4 Clinical Research Department, Diagnostica Stago, Gennevilliers, France

## Abstract

Venous thromboembolism (VTE) is a common complication in newly diagnosed symptomatic multiple myeloma (NDMM) patients. We explored cellular and plasma hypercoagulability in NDMM patients to identify relevant biomarkers that can be used in combination with clinical factors in the development of a risk assessment model (RAM) for VTE. Untreated patients (*n* = 144) with NDMM were prospectively enrolled, baseline biomarkers prior to anti-myeloma treatment and thromboprophylaxis initiation were obtained. These were compared against values in a group of healthy individuals with similar age and sex distribution. The primary study end point was symptomatic VTE occurrence. At 12-month follow-up cumulative VTE rate was 10.4%. NDMM patients showed biological signs of cellular and plasma hypercoagulability and endothelial cell activation. Procoagulant phospholipid clotting time (Procoagulant-PPL) was shorter, P-selectin levels lower and thrombin generation attenuated overall compared to healthy subjects. Longer Procoag-PPL^®^, lower endogenous thrombin potential (ETP), and higher levels of tissue factor pathway inhibitor (TFPI) were associated with VTE occurrence. Multivariate analysis showed that Procoag-PPL^®^ and ETP were independent risk factors for VTE. We conclude that Procoag-PPL^®^ and ETP can be prospectively incorporated into a RAM for VTE in MM in combination with clinical and disease risk factors.

## Introduction

Multiple myeloma (MM) is among malignancies that significantly increase the risk of venous thromboembolism (VTE)^1^. The incidence of VTE in MM patients is estimated at 8–22 per 1000 person years and approximately 10% of newly diagnosed MM patients (NDMM) will develop VTE^2–4^.The risk of VTE is linked to patient-related clinical factors, type of anti-myeloma therapy, and disease-specific mechanisms^5,6^. The choice of therapy has been shown to affect the risk of VTE to a large extent. The rate of VTE is about 1–2% in patients who receive conventional therapies, such as melphalan and prednisone. Immunomodulatory drug monotherapy (IMiD) is associated with a slightly increased rate of 3–4% at initial diagnosis. The effect on VTE rate is multiplied when IMiDs are combined with corticosteroids and chemotherapy and incidence can be as high as 26%^3,7^. The International Myeloma Working Group (IMWG) statement and the European Guidelines propose a VTE risk assessment method to guide pharmacological thromboprophylaxis application^8–14^. Despite administration of thromboprophylaxis as per guidelines, the risk of residual VTE remains as high as 10%, revealing that the available RAM is suboptimal^3,15^.

The prospective, observational study ROADMAP-MM-CAT (PROspective Risk Assessment anD bioMArkers of hypercoagulability for the identification of patients with Multiply Myeloma at risk for Cancer-Associated Thrombosis) enrolled treatment naïve symptomatic NDMM patients. Symptomatic VTE was the clinical outcome. It aimed to explore the profile of blood borne hypercoagulability in chemotherapy naïve patients with symptomatic MM and to identify relevant biomarkers of hypercoagulability that can be prospectively used in combination with variables related with MM and clinical predictors of VTE risk to guide thromboprophylaxis.

## Methods

### Study design and participants

The study was an investigator initiated prospective non-interventional trial. Newly diagnosed, treatment naïve symptomatic patients with MM (based on 2014 IMWG Criteria)^16^ were diagnosed or referred to the Department of Clinical Therapeutics (Alexandra Hospital, Athens, Greece) from June 2014 to June 2017. Exclusion criteria included age less than 18 years, recent (<6 months) episode of VTE and active anticoagulant treatment (for any indication) prior to enrollment in the study, ongoing pregnancy, recent hospitalization for surgical illness, active malignancy other than MM, surgery in the preceding 3 months. All patients provided written informed consent and all patients received anti-myeloma treatment according to institutional practice.

### Procedures

Α standardized clinical research form (CRF) was completed for all patients enrolled in the study at baseline and at 3, 6, and 12 months post-treatment initiation, which assessed disease parameters, VTE risk factors, status of disease, ongoing treatments, hematology, and renal and liver blood function tests were recorded most of which will be presented elsewhere. The term “cardiovascular risk factor” included the presence of any of the following; hypertension, hyperlipidemia, ischemic heart disease, smoking, an arrhythmia, or diabetes. Renal function was assessed using Cockcroft–Gault formula (ml/min). Performance status (PS) was assessed according to the Eastern Cooperative Oncology Group (ECOG) classification. At diagnosis, patients were classified according to the international staging system for MM as stage I, II, or III. Patients were classified as having high-risk cytogenetics at diagnosis if bone marrow fluorescence in situ hybridization analysis was positive for t(4;14), t(14;16), or del17q cytogenetic abnormalities. The presence of lytic bone disease was assessed with whole body computerized tomography (WBCT) scans. Magnetic resonance imaging (MRI) of the thoracic and lumbar spine was used to assess the pattern of bone marrow infiltration (normal, focal pattern, salt-and-pepper, or diffuse).

Patients were routinely assessed for DVT with Doppler ultrasound of the lower limbs at inclusion. VTE risk assessment and choice of thromboprophylaxis post enrollment in the study were along IMWG 2014 recommendations^8–10^. High-risk patients (IMiDs plus > 1 VTE risk factor) received tinzaparin 4500 anti-Xa IU sc od, moderate risk patients (IMiDs plus no risk factors or non-IMiDs with moderate risk for VTE) received aspirin 100 mg po od and low-risk patients (no or very low-risk plus non-IMiD therapy) did not receive any thromboprophylaxis. The duration of thromboprophylaxis depended on treatment duration and type. Thromboprophylaxis choice was not considered to be an intervention as it is a part of the standard clinical care provided by the institution for this population.

### Molecular and functional blood analysis

Blood samples were routinely obtained by atraumatic antecubital venipuncture at baseline and collected in Vacutainer® tubes (5 ml tubes, containing 0.109 mol/L trisodium citrate—1 volume trisodium citrate to 9 volumes blood). They were used to extract platelet-poor plasma (PPP) by double centrifugation at 2000 × *g* for 20 minutes at room temperature and plasma aliquots were stored at −80 °C until assayed.

Blood samples were centralized to the core laboratory at Thrombosis Center, Service d’Hématologie Biologique, Tenon University Hospital, Paris. Procoagulant phospholipid-dependent clotting time *(Proag-PPL)* was measured with STA^®^Procoag-PPL, according to the manufacturer’s instructions^17,18^. The levels of factor VIIa (Staclot^®^ VIIa-rTF), factor V (FV), antithrombin (AT), fibrin monomers (FM), free TFPI, and D-Dimers were measured with commercially available assays according to the manufacturer’s instructions, on a STA-R® analyzer. The assays and the analyzer were purchased from Diagnostica Stago, Asnières France. Tissue Factor activity (TFa) in PPP was measured as previously described^17,19,20^. The inter- and intra-assay coefficients of variation were 7% and 5%, respectively. Plasma levels of P-selectin and heparanase were measured with ELISA Kits from Cusabio Biotech (CliniSciencies, France) and R&D Systems (Lille, France), respectively. Thrombin generation was assessed in samples of PPP with the TF 5 pM PPP-Reagent^®^ on Calibrated Automated Thrombogram (Stago, France). The following parameters of thrombogram were analyzed: (a) lag-time that indicates the initiation phase of thrombin generation, (b) time to reach maximum concentration of thrombin (ttPeak), (c) maximum concentration of thrombin (Peak), d) mean rate index (MRI) of the propagation phase of thrombin generation calculated by the formula: Peak/(ttPeak – lag-time) and expressed in nM/min and e) endogenous thrombin potential (ETP) that shows the integral enzymatic activity of thrombin as described elsewhere^21–23^.

Biomarkers measured in the cohort of MM patients were compared against values previously measured in a group of healthy individuals with similar age and sex distribution, not taking any medication for at least 1 month before blood sampling.

The normal values of the studied biomarkers were defined in the control group and were compared to the corresponding normal reference range used by our laboratory. These normal ranges have been established according to the requirements for the good quality of laboratory practice by performing the tests in healthy individuals representative of the general population regarding age, sex, ethnicity, and BMI.

### Outcomes

The primary study end point was symptomatic VTE, objectively confirmed by at least one of the following methods: color Echo-Doppler, computerized tomography, magnetic resonance imaging angiography, scintigraphy, or computerized tomography scan. Symptomatic VTE included deep vein thrombosis (DVT), pulmonary embolism (PE) or both (DVT and PE), superficial vein thrombosis located at distance of <3 cm from the saphenofemoral junction (SVT), central venous catheter (CVC) thrombosis or upper limb vein thrombosis (not related to the CVC) or vein thrombosis of rare localization (i.e. splanchnic vein or cerebral vein thrombosis). Patients with incidental VTE (asymptomatic venous thrombosis of the lower limb detected at baseline/screening Doppler ultrasound) were not included in the analysis.

### Statistics

Continuous variables are described as mean ± standard deviation and categorical variables as frequency and percentage. In view of the deviation from normality (as evidenced by the Shapiro-Wilk test), the comparison of biomarker levels between MM patients and healthy individuals was performed using the Mann-Whitney-Wilcoxon text for independent samples. Concerning the intercorrelations between biomarkers in MM patients, Spearman’s rank correlation coefficients were estimated. At the univariate analysis the level of statistical significance was set at 0.05.

Regarding the associations between VTE and biomarkers, the latter were converted to binary variables through Receiver Operating Characteristic (ROC) curve analysis; the selection of cut-off levels was based on the maximization of Youden’s index. Subsequently, multivariate logistic regression analysis was performed with VTE as the dependent variable; biomarker variables proven significant at the univariate logistic regression analysis were examined as possible independent variables. Using a stepwise procedure, at the final multivariate logistic model all variables with *p*-value less than 0.10 were retained; the area under the ROC curve (AUC) was estimated to describe the fit of the multivariate model. Data were analyzed using the STATA/SE version 13 statistical software (Stata Corp., College Station, TX, USA).

### Ethics

The protocol of the study was in accordance with the commitment of the Helsinki declaration and was approved by the institutional ethics committee (Hospital Ethics and Scientific Committee). All patients provided informed written consent before enrollment in the study.

## Results

### Study population

A total of 144 eligible patients with NDMM were enrolled in the study from June 2014 to June 2017. No patients were lost on follow-up or excluded from analysis due to missing data. The demographics and clinical characteristics of the patients at the time of inclusion are summarized in Table 1. Median age was 66.0 ± 11.6 (36–86) years and 53% of the population was male. Cardiovascular risk factors were present in 34% of patients. At inclusion all patients were naïve regarding any anti-myeloma treatment. Disease stage in the population was distributed as follows: 32% were ISS-I, 23% ISS-II, and 45% ISS-III. Bone disease was present in 71% of patients and 27% had high-risk cytogenetics. Proteasome inhibitor (PI) based therapy was given to 64% of patients, immunomodulatory drug (IMiD) based therapy in 32%, and 4% received other regimens.

**Table 1:** Baseline demographic, clinical, and biological characteristics of multiple myeloma patients

Patients’ clinical characteristics	
Age (years)	66.0 ± 12.0 (36–86)
Male/female	76/68 (53%/47%)
BSA (m^2^)	1.85 ± 0.20 (1.46–2.50)
BMI (kg/m^2^)	25.9 ± 5.0 (17.2–44.8)
ISS stage - *n* (%)	
I	46 (32%)
II	33 (23%)
III	65 (45%)
MM type - *n* (%)	
IgA	37 (26%)
IgG	80 (55.5%)
κLC	18 (12.5%)
λLC	9 (6.0%)
Anti-myeloma treatment - *n* (%)	
PI-based	92 (64%)
IMiD-based	46 (32%)
Other	6 (4%)
ECOG performance status - *n* (%)	
0	57 (44.7)
1	59 (41)
2	23 (16)
3	4 (2.8)
4	1 (0.7)
Dialysis at diagnosis - *n* (%)	14 (10%)
Bone disease present - *n* (%)	102 (71%)
High-risk cytogenetics- *n* (%)	27 (19%)
Comorbidities and VTE risk factors not related with the cancer - *n* (%)	
Active pulmonary disease	13 (9%)
CV risk factors	110 (76.4%)
EPO use	50 (35%)
GFR < 30 ml/min	22 (15%)
Thromboprophylaxis after enrollment in the study - *n* (%)	
None	47 (33)
Aspirin	74 (51.0)
LMWH (tinzaparin)	23 (16)
Patients’ biological data (mean ± SD; range)	
β2-microglobulin (mg/dl)	8.0 ± 8.7 (0.06–48.5)
M-peak (g/dl)	2.9 ± 2.3 (0–9)
U-peak (mg/24h)	356 ± 846 (0–6667)
Bone marrow infiltration (%)	61.4 ± 27.0 (0–100)
Total protein (g/dl)	8.6 ± 2.1 (5.1–14.3)
Creatinine (mg/dl)	1.85 ± 3.0 (0.47–28.0)
Urea (mg/dl)	58.1 ± 42.0 (5–276)
GFR (ml/min)	73.0 ± 43.0 (4.2–230.0)
LDH (U/l)	196 ± 96 (70–789)
ALT (U/l)	24.3 ± 21.0 (6–162)
AST (U/l)	24.1 ± 24.0 (6–178)
Albumin (g/dl)	3.8 ± 0.7 (2.1–6.8)
Calcium (mg/dl)	9.7 ± 1.1 (6.7–13.4)
Hb (g/dl)	10.5 ± 2.0 (7.0–17.5)
White blood cell count (×10^6^/μl)	6.8 ± 3.0 (0.48–18.8)
Neutrophils (×10^6^/μl)	4.3 ± 2.3 (0.2–12.6)
Platelets (×10^3^/μl)	256 ± 125 (26–879)

*CV* cardiovascular, *EPO* erythropoetin, *ISS* International Staging system per ISS, *VTE* venous thromboembolism, *LMWH* low molecular weight heparin, *PI* proteasome inhibitor based, *IMiD* immunomodulatory drug based, *MM* multiple myeloma, *BMI* body mass index, *BSA* body surface area, *M-peak* serum monoclonal protein, *U-peak* urine monoclonal protein, *Hb* hemoglobin, *LDH* lactate dehydrogenase, *GFR* glomerular filtration rate, *ALT* alanine aminotransferase, *AST* aspartate aminotransferase, *ECOG* Eastern Cooperative Oncology Group

After enrollment in the study and based on current thromboprophylaxis guidelines, current risk stratification, and standard clinical care provided by the institution, 33% of patients did not receive any thromboprophylaxis, 51% received aspirin 100 mg o.d., and 16% received tinzaparin 4500 anti-Xa IU s.c. o.d. Median follow-up time was 15.5 months (27 days–35 months). During follow-up 37 patients died.

### Follow-up and VTE

The overall rate of symptomatic VTE during follow-up was 10.4% (*n* = 15 out of 144 patients). Nine out of 15 events (60%) occurred within 3 months from treatment initiation. Six of these patients did not receive any thromboprophylaxis; six patients were on aspirin at the time of the event and three were on LMWH. Out of the patients that received IMiD-based therapy 74% were on aspirin, 14% were on LMWH, and 12% received no thromboprophylaxis. Out of the patients that received PI-based treatment 54% received no thromboprophylaxis, 36% were on aspirin, and 10% received LMWH. The rate of VTE did not differ significantly between patients who received thromboprophylaxis and those who did not. The rate of VTE was not significantly different between patients who received IMiD-based (11.4%) treatment and patients on other therapy (10%). Almost half of the events were distal DVT (46.7%) and 13.3% were pulmonary embolism. Analytical data on patients with VTE are shown in Table 2.

**Table 2:** Venous thromboembolism events among study population

Patients’ identification #	Sex	Age	Localization	Time of event from diagnosis (days)	Disease status at follow-up	Thrombo-prophylaxis	Anti-myeloma treatment
1A	Μ	50	IJV thrombosis post CVC insertion	150	PR	No	ASCT
2A	F	46	IJV thrombosis post CVC insertion	90	VGPR	No	ASCT
3A	M	40	Superficial UL vein thrombosis	90	PR	Aspirin	RAD
4A	F	76	Superficial LL vein thrombosis	60	PR	No	VMP
5A	M	78	Distal DVT	45	PR	LMWH	CTD
6A	M	81	Distal DVT	45	PR	Aspirin	VMP
7A	M	68	Distal DVT	15	PR	No	VCD
8A	M	55	PE	180	PD	Aspirin	RD
9A	F	88	Mesenteric vein thrombosis	90	SD	LMWH	CTD
10A	M	62	Distal DVT	360	VGPR	Aspirin	RD
11A	F	73	Distal DVT	270	PR	LMWH (prior to event)	RD
12A	M	71	Distal DVT	330	PR	Aspirin	RD
13A	M	43	IJV thrombosis–CVC insertion	135	PR	No	ASCT
14A	F	81	Distal DVT	30	SD	Aspirin	RD
15A	M	59	PE	10	Not evaluable	None	None

*M* male, *F* female, *DVT* deep vein thrombosis of the lower limb,*CVC* central venous catheter insertion, *IJV* internal jugular vein, *LL* lower limb, *UL* upper limb, *PR* partial response, *VGPR* very good partial response, *PD* progressive disease, *SD* stable disease, *ASCT* autologous stem cell transplant, *RAD* revlimid, adriamycin, and dexamethasone, *VMP* velcade, melphalan, and prednisone, *CTD* cyclophosphamide, thalidomide, and dexamethasone, *VCD* velcade, cyclophosphamide, and dexamethasone, *RD* revlimid and dexamethasone

### Biomarkers of hypercoagulability and VTE risk

#### Hypercoagulability at diagnosis of multiple myeloma prior to treatment initiation

At inclusion, patients showed significantly increased levels of TFa, FVIIa, D-Dimers and FM, and significantly shorter Procoag-PPL^®^ as compared to the group of healthy individuals. Levels of P-selectin and TM were significantly lower in patients as compared to healthy individuals. The levels of heparanase were not significantly different in the group of patients as compared to the healthy individuals. Overall thrombin generation was attenuated in patients compared to healthy individuals. Lag-time and ttPeak were significantly increased and Peak, MRI, and ETP were significantly lower as compared to the group of healthy individuals (Figs.^1^,^2^,^3^, Table 3). The TFPI and TM were positively correlated with chronometric parameters of thrombogram and negatively correlated with ETP and Peak. TFPI was also positively correlated with PPL-ct and TM. Results are presented in Table 4 and should be deemed explorative in view of multiple comparisons tested simultaneously.

**Fig. 1:**
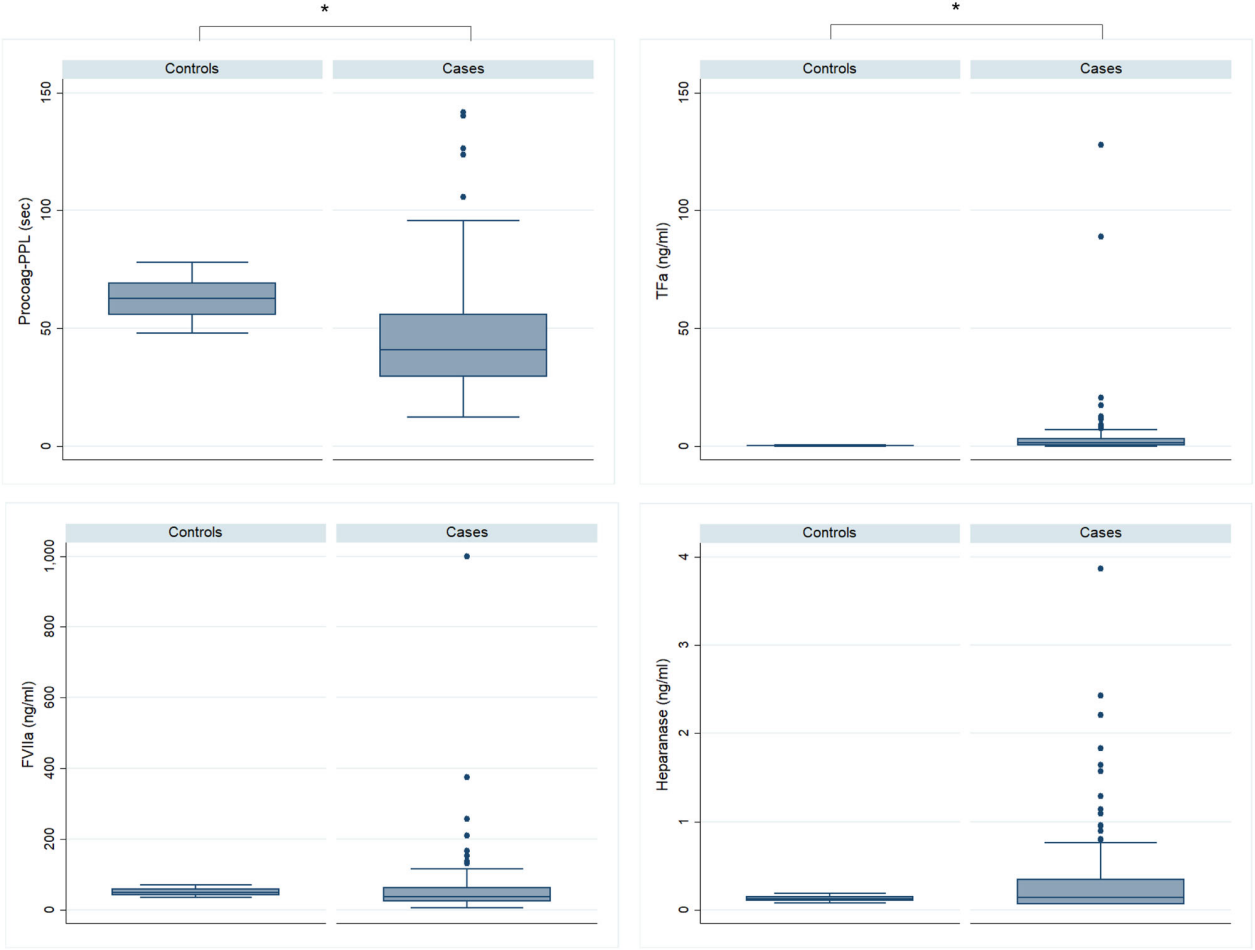
Boxplots showing the biomarkers in MM patients versus controls. PPL-ct procoagulant phospholipid dependent clotting time, TFa tissue factor activity, FVIIIa activity of factor VIII, *p < 0.0001

**Table 3:** Profile of hypercoagulability in patients at diagnosis of MM prior to treatment initiation

	Normal reference range	Healthy subjects (*n* = 30)	MM (*n* = 144)	*p*
Cellular-derived hypercoagulability				
Procoag-PPL (s)	42–85	62.8 ± 8.6	45.6 ± 22.6	<0.0001
TFa (ng/ml)	0.02–0.45	0.26 ± 0.13	3.97 ± 13.10	<0.0001
Heparanase (ng/ml)	0.08–0.16	0.13 ± 0.03	0.34 ± 0.52	0.476
TM (%)	70–120	90 ± 18	39.25 ± 68.1	<0.005
P-selectin (μg/ml)	82–42	62.66 ± 103.91	38.12 ± 31.78	<0.0001
TFPI (ng/ml)	15–26	18 ± 4	31 ± 18.5	0.02
Blood coagulation factors and natural inhibitors				
FVIIa (U/ml)	73–29	50.9 ± 10.6	74.1 ± 147.6	0.022
FV (%)	70–120	90 ± 12	78 ± 11	0.23
AT (%)	70–120	92 ± 12.0	95.4 ± 17.7	<0.005
In vivo fibrin formation/lysis				
D-Dimers (μg/ml)	<0.50	0.31 ± 0.08	1.80 ± 3.41	<0.0001
FM (μg/ml)	0.5–5.50	2.5 ± 0.5	14.29 ± 31.8	<0.0001
Thrombogram parameters				
Lag-time (min)	2.1–3.8	2.53 ± 0.43	4.20 ± 2.16	<0.0001
ttPeak (min)	4.0–6.6	5.28 ± 0.73	7.33 ± 2.76	<0.0001
Peak (nM)	222–330	287.8 ± 35.7	214.4 ± 80.1	<0.0001
MRI (nM/min)	60–120	109.9 ± 24.5	80.2 ± 45.7	<0.0001
ETP (Mxmin)	1600–1178	1496.8 ± 191.4	1181.8 ± 398	<0.0001

*Procoag-PPL* procoagulant phospholipid-dependent clotting time, *TFa* tissue factor activity, *TM* thrombomodulin activity, *TFPI* tissue factor pathway inhibitor, *FVIIa* activated factor VII, *FV* factor V, *ATIII* antithrombin, *FM* fibrin monomers, *ttPeak* time to peak of thrombin, *MRI* mean rate index of thrombin generation, *ETP* endogenous thrombin potential

*p*-values derived from Mann–Whitney–Wilcoxon test for independent samples (comparison of patients versus healthy individuals)

**Table 4:** Spearman’s rank correlation coefficient (*p*-values in brackets) showing the intercorrelations between hypercoagulability biomarkers and M-peak assessed in patients before treatment administration

M-peak																	
MRI																	**−0.184 (0.028)**
ttPeak																+0.044 (0.614)	−0.052 (0.549)
Peak															**+0.606 (<0.0001)**	+0.043 (0.710)	−0.171 (0.137)
ETP														**+0.292 (0.032)**	−0.036 (0.729)	**+0.239 (0.020)**	**−0.367 (0.0003)**
Lag-time													+0.220 (0.104)	**+0.314 (0.022)**	−0.069 (0.499)	+0.098 (0.336)	**+0.219 (0.029)**
FM												+0.078 (0.451)	−0.065 (0.541)	**+0.265 (0.020)**	+0.139 (0.112)	+0.137 (0.105)	+0.027 (0.755)
D-di											−0.163 (0.091)	+0.073 (0.521)	+0.101 (0.445)	**−0.228 (0.046)**	−0.045 (0.653)	+0.017 (0.862)	**−0.207 (0.031)**
ATIII										**+0.362 (0.0001)**	−0.107 (0.205)	+0.056 (0.581)	**−0.205 (0.047)**	−0.160 (0.167)	+0.107 (0.214)	+0.131 (0.118)	−0.157 (0.061)
FV									−0.111 (0.187)	**−0.197 (0.040)**	**+0.211 (0.012)**	**+0.482 (<0.0001)**	**+0.212 (0.039)**	**+0.357 (0.002)**	+0.159 (0.064)	**+0.241 (0.004)**	+0.062 (0.463)
FVIIa								**+0.243 (0.013)**	−0.078 (0.429)	+0.022 (0.859)	+0.102 (0.308)	**+0.435 (0.0003)**	+0.072 (0.537)	+0.099 (0.554)	**+0.281 (0.005)**	+0.099 (0.316)	+0.086 (0.383)
TFPI							+0.113 (0.264)	**+0.284 (0.0008)**	**+0.258 (0.002)**	**+0.232 (0.019)**	−0.054 (0.538)	**+0.560 (<0.0001)**	−0.099 (0.348)	−0.079 (0.504)	−0.077 (0.383)	+0.090 (0.298)	**+0.274 (0.001)**
P-sel						**−0.254 (0.003)**	−0.122 (0.231)	−0.014 (0.876)	−0.013 (0.876)	−0.005 (0.962)	+0.146 (0.093)	**−0.209 (0.043)**	−0.015 (0.886)	+0.176 (0.133)	+0.121 (0.169)	+0.105 (0.224)	**−0.219 (0.010)**
TMa					**+0.794 (<0.0001)**	**−0.358 (<0.0001)**	−0.092 (0.364)	−0.019 (0.823)	+0.125 (0.147)	−0.066 (0.509)	+0.163 (0.060)	**−0.283 (0.006)**	−0.119 (0.258)	**+0.260 (0.026)**	+0.166 (0.059)	+0.112 (0.193)	**−0.262 (0.002)**
Hep				**−0.605 (<0.0001)**	**−0.358 (<0.0001)**	**+0.886 (<0.0001)**	+0.125 (0.220)	**+0.177 (0.039)**	**+0.238 (0.005)**	**+0.279 (0.004)**	−0.050 (0.569)	**+0.501 (<0.0001)**	−0.049 (0.644)	−0.204 (0.082)	−0.090 (0.311)	+0.034 (0.695)	**+0.258 (0.002)**
TFa			**−0.723 (<0.0001)**	**+0.953 (<0.0001)**	**+0.652 (<0.0001)**	**−0.427 (<0.0001)**	−0.100 (0.324)	−0.011 (0.903)	+0.022 (0.802)	−0.164 (0.098)	+0.133 (0.125)	**−0.311 (0.002)**	−0.073 (0.489)	**+0.233 (0.045)**	+0.145 (0.099)	+0.097 (0.259)	**−0.224 (0.008)**
PPL		**+0.261 (0.002)**	**−0.409 (<0.0001)**	+0.147 (0.087)	+0.101 (0.241)	**−0.318 (0.0002)**	−0.144 (0.145)	−0.009 (0.913)	**−0.369 (<0.0001)**	**−0.190 (0.048)**	−0.075 (0.378)	−0.189 (0.061)	+0.113 (0.274)	−0.053 (0.647)	−0.032 (0.713)	−0.070 (0.406)	−0.088 (0.296)
	PPL	TFa	Hep	TMa	P-sel	TFPI	FVIIa	FV	ATIII	D-di	FM	Lag-time	ETP	Peak	ttPeak	MRI	M-peak

Bold cells denote intercorrelations with *p* < 0.05

### Predictors of VTE

ROC analysis defined cutoffs for biomarkers and univariate analysis was performed. The analysis showed that patients with Procoag-PPL^®^ ≥47 had a 3.49 times higher risk of VTE compared to patients with Procoag-PPL^®^ <47s (OR = 3.49, 95% CI: 1.13–10.82, *p* = 0.030). In addition, patients with ETP ≥ 1087 nM×min versus patients with ETP < 1087 nM×min had significantly lower risk of VTE (OR = 0.25 95% CI: 0.07–0.83, *p* = 0.024). Finally, patients with TFPI ≥ 39 versus patients with TFPI < 39 had a 7.75 higher risk of VTE (OR = 7.74 95% CI: 1.51–39.70, *p* = 0.014). (Table 5)

**Table 5:** Univariate logistic regression analysis evaluating associations between the examined biomarkers and VTE

	Compared categories	OR (95% CI)	*p*
Cellular-derived hypercoagulability			
Procoag-PPL (s)	≥47.0 vs. <47.0	3.49 (1.13–0.82)	0.030
TFa (ng/ml)	≥0.03 vs. <0.03	0.49 (0.09–2.50)	0.389
Heparanase (ng/ml)	≥0.68 vs. <0.68	Not estimable due to zero events in the upper category	0.215^F^
TMa (%)	≥42.0 vs. <42.0	4.93 (0.97–24.99)	0.054
P-selectin (pg/ml)	≥46700 vs. <46700	2.69 (0.71–10.26)	0.147
TFPI (ng/ml)	≥39.0 vs. <39.0	7.75 (1.51–39.70)	0.014
Blood coagulation factors and natural inhibitors			
FVIIa (ng/ml)	≥56.8 vs. <56.8	0.34 (0.07–1.59)	0.172
FV (%)	≥103 vs. <103	0.15 (0.02–1.18)	0.071
ATIII (%)	≥87 vs. <87	2.33 (0.50–10.84)	0.282
In vivo thrombin generation			
D-Dimers (μg/ml)	≥2.1 vs. <2.1	2.52 (0.82–7.69)	0.105
FM (μg/ml)	≥8.4 vs. <8.4	2.07 (0.61–6.95)	0.241
Thrombogram parameters			
Lag-time (min)	≥6.5 vs. <6.5	Not estimable due to zero events in the upper category	0.612^F^
ETP (M×min)	≥1087 vs. <1087	0.25 (0.07–0.83)	0.024
Peak (nM)	≥253.0 vs. <253.0	1.50 (0.49–4.61)	0.479
ttPeak (min)	≥10 vs. <10	Not estimable due to zero events in the upper category	0.364^F^
MRI (nM/min)	≥121 vs. <121	1.40 (0.36–5.49)	0.625

F: *p*-value derived from Fisher’s exact test. The cutoff levels were set on the basis of the respective ROC curves. *PPL-ct* procoagulant phospholipid dependent clotting time, *TFa* tissue factor activity, *TM* thrombomodulin activity, *TFPI* tissue factor pathway inhibitor, *FVIIa* activity of factor VII, *FV* factor V, *ATIII* anti-thrombin, *FM* fibrin monomer, *ETP* the endogenous thrombin potential, *Peak* the peak concentration of thrombin, *ttPeak* time to reach the peak concentration of thrombin, *MRI* mean rate index of thrombin generation

*p*-values derived from Mann–Whitney–Wilcoxon test for independent samples

### Multivariate logistic regression

The type of thromboprophylaxis (none; aspirin; LMWH) was not associated with VTE risk (*p* = 0.535, Fisher’s exact test). Therefore, thromboprophylaxis was not entered into the multivariate logistic regression analysis. Following systematic testing by univariate analysis of all biomarkers we identified the significant predictors for use in a multivariate model. Multivariate logistic regression analysis demonstrated that ETP <1087 nM×min versus ≥1087 nM×min (OR = 4.04, 95% CI: 1.18–13.84, *p* = 0.026) and Procoag-PPL^®^ ≥47 versus <47 s (OR = 3.01, 95% CI: 0.93–9.78, *p* = 0.066) were independently associated with VTE occurrence.

**Fig. 2:**
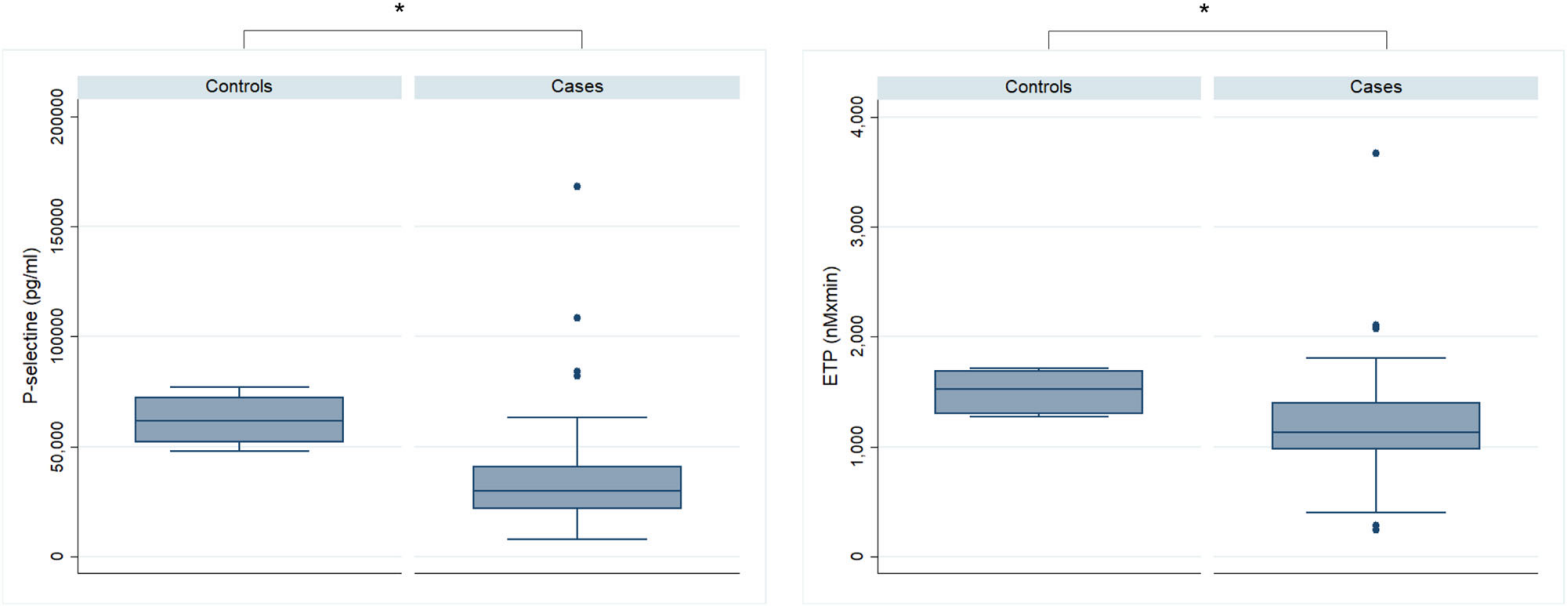
Boxplots showing the biomarkers in MM patients versus controls. ETP the endogenous thrombin potential. * *p* < 0.0001

This multivariate logistic regression model corresponded to the following equation:

log(odds for VTE) = −3.51 + (1.40 × ETP_binary) + (1.10 × Procoag-PPL^®^_binary)

Based on the equation above, a score was formulated where the dependent variable is the log(odds for VTE) and the binary predictors: 1 for Procoag-PPL^®^ ≥47 and 1 for ETP < 1087 nM×min or 0 for Procoag-PPL^®^ <47 s and ETP ≥ 1087 nM×min, respectively. The AUC of the ROC analysis was 0.73. Patients were stratified at high or intermediate/low-risk group; the optimal cut-off level in the aforementioned score was equal to −2.11. The rate of VTE was 5% in the intermediate/low-risk group and 17.5% in the high-risk group. The rate of VTE was not significantly associated with the type of thromboprophylaxis in either the intermediate/low-risk group (*p* = 0.62, Fisher’s exact test) or the high-risk group (*p* = 0.588, Fisher’s exact test). The sensitivity and the specificity of the score were 71.4% and 61.8%, respectively. According to the Hosmer–Lemeshow test, a value of *p* = 0.858 showed that the model was well calibrated.

**Fig. 3:**
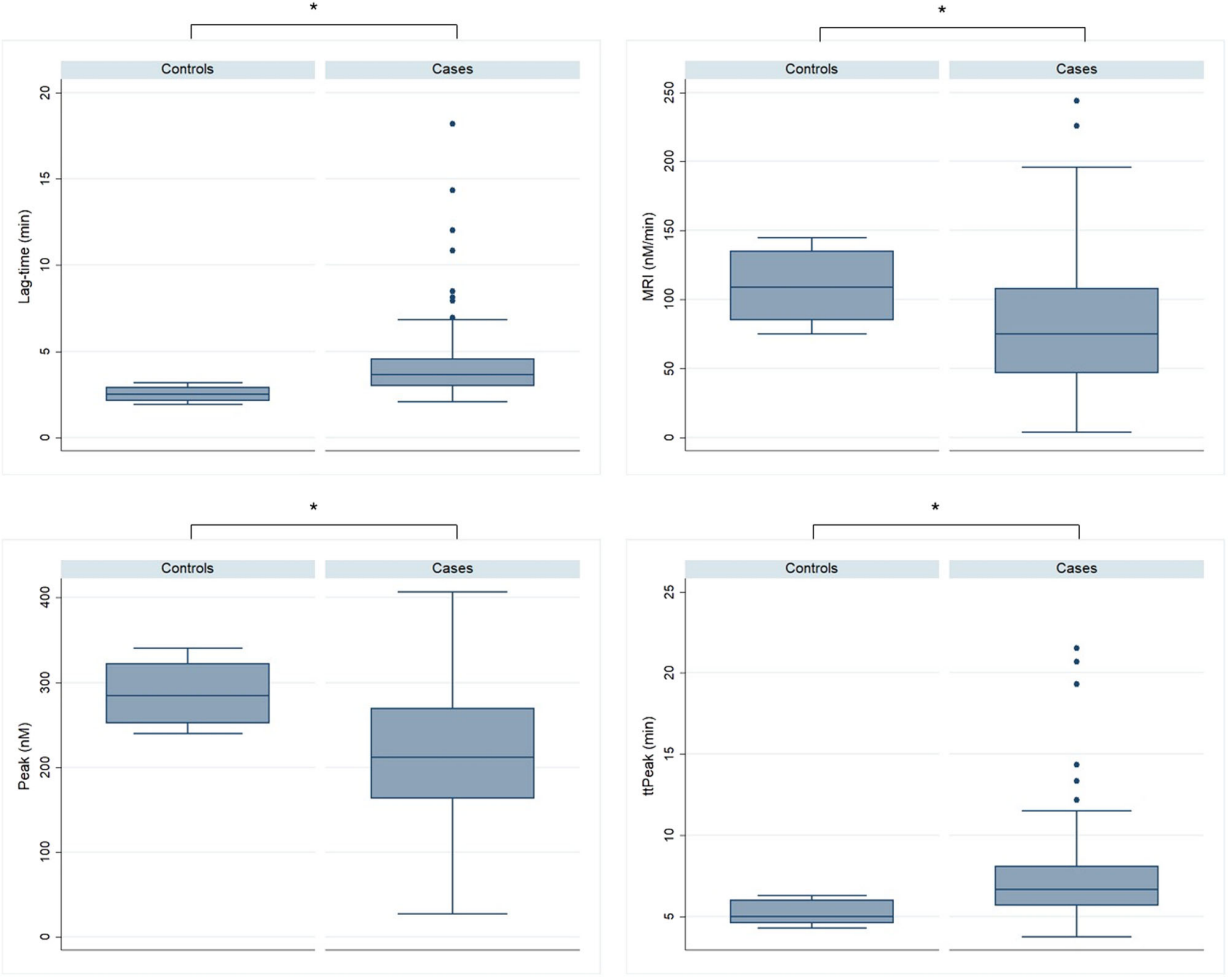
Boxplots showing the biomarkers in MM patients versus controls. MRI mean rate index of thrombin generation, ttPeak time to reach the peak concentration of thrombin. *p < 0.0001

## Discussion

The prospective observational ROADMAP-MM-CAT study explored the complex coagulation profile of newly diagnosed, chemotherapy naïve patients with symptomatic MM and identified clinically relevant biomarkers for use in VTE risk stratification. The study provides biological evidence linking cell derived hypercoagulability with MM disease and VTE risk. The design of the ROADMAP-MM-CAT study led to derivation of a new score, based on biomarkers of hypercoagulability, for accurate stratification of patients as high and intermediate/low-risk for VTE. In the studied cohort of NDMM patients, the overall rate of symptomatic VTE was 10.4% and this is in accordance with recent literature^12–14^. In agreement with the literature, the rate of VTE was highest during the first 4 months following diagnosis and distal DVT was the most frequent localization of VTE^3^. VTE events occurred across all risk groups for VTE despite administration of thromboprophylaxis based on current recommendations. The study design did not allow detection of potential differences in efficacy and safety of thromboprophylaxis with aspirin or LMWH in patients stratified as intermediate/low- or high-risk for VTE. As a result, the data confirm that the residual rate of symptomatic VTE events remains high and underline the need for an improved tool for the selection of patients eligible for thromboprophylaxis and an alternative antithrombotic strategy^8,9^.

The study was prospective and the clinical features of the derivation cohort responded to the principal generalizability criteria for risk assessment tools^24–26^. Moreover, it included routine pre-entry VTE status with Doppler echography and vigorous assessment of VTE risk. A large number of hypercoagulability biomarkers were assessed before treatment administration. Among them, procoagulant phospholipid-dependent clotting time (Procoag-PPL^®^), endogenous thrombin generation potential (assessed in PPP with the Calibrated Automated Thrombogram-Thrombinoscope^®^ assay using the 5 pM TF PPP-Reagent^®^), and the levels of TFPI were significant predictors of VTE risk. Multivariate analysis led to derivation of the new ROADMAP-CAT-MM score which combines Procoag-PPL® clotting time and ETP and accurately stratifies patients into high and intermediate/low-risk for VTE. The sensitivity and the specificity of the new score are 71.4% and 61.8%, respectively and the AUC of the ROC analysis is 0.73.

The Procoag-PPL^®^ clotting time is correlated with the concentration of procoagulant microparticles derived from platelets or other cells^27^. All patients had shorter Procoag-PPL^®^ clotting time than the lower normal levels of this test indicating that platelet and/or endothelial cell activation is linked with MM disease. Patients had low-levels of P-selectin (a marker of platelet activation) reflecting a state of chronic platelet activation what has been previously described as “exhausted platelet syndrome”^28,29^. This finding further corroborates the concept that sustained platelet activation is part of the disease process in MM. In addition, the increased levels of TFa and TFPI in patients (15-fold and 1.7-fold, respectively, as compared to those in healthy individuals) reflect systematic endothelial cell activation. These proteins have substantially different roles in the blood coagulation mechanism. TF is the initiator of coagulation whereas TFPI belongs to the natural anticoagulant system. Nevertheless, both proteins are synthesized and released by activated endothelial cells^30,31^. Consequently, the increase of these biomarkers, along with the increase of FVIIa, should be interpreted as indicators of endothelial cell activation.

Patients also showed an unexpected attenuation of thrombin generation in plasma as lag-time and ttPeak were significantly prolonged, whereas Peak, MRI, and ETP were significantly lower as compared to the group of healthy individuals. This finding may seem a paradox but is in agreement with the data published by Legendre et al^24^. A methodological approach is required for the interpretation of this finding. Thrombogram-Thrombinoscope^®^ assay is performed with exogenously added optimal concentrations of TF (5 pM) and procoagulant phospholipids (4 μM). Thus, the sensitivity of the test to variations of plasma concentrations of TF or procoagulant phospholipids is limited. In contrast, under the methodological conditions used in this study, the test is sensitive to the variations of TFPI and/or TM levels in plasma (data from in vitro experiments not shown). In the cohort of studied MM patients correlation analysis showed that the attenuation of thrombin generation is related to increased plasma concentration of TFPI and TM. Accordingly, in patients with newly diagnosed MM, the attenuation of thrombin generation should be interpreted as a reflection of endothelial cell activation rather than as an indicator of plasma hypercoagulablity; i.e. an imbalance between clotting factors and natural coagulation inhibitors. The increased levels of fibrin monomers and D-Dimers found in NDMM patients’ plasma document an environment of increased plasma hypercoagulability, which is in accordance with previous studies^26^,^32^.

The prospective design of the ROADMAP-MM-CAT study allowed identification of biomarkers of hypercoagulability as predictors for VTE risk. Patients with Procoag-PPL^®^ clotting time ≥47s had a 3.5-fold higher risk of VTE as compared to those with a Procoag-PPL^®^ clotting time shorter than this cutoff. Although this finding seems unexpected, we could assume that it lies in the same context with P-selectin decrease and eventually reflects the presence of exhausted platelets. Patients with ETP ≥ 1087 nM×min versus patients with ETP < 1087 nM×min had a lower VTE risk. In addition, patients with TFPI ≥ 39 ng/ml versus those with TFPI < 39 ng/ml had a 7.75 higher VTE risk (OR = 7.74, 95% CI: 1.51–39.70). This analysis further supports the concept that TFPI levels increase in plasma and that thrombin generation attenuation reflects endothelial cell activation rather than a real downregulation of plasma hypercoagulable state.

The feasibility of the new ROADMAP-CAT-MM score for VTE risk is a challenge for the new strategy for the identification of multiple myeloma patients eligible for pharmacological thromboprophylaxis. Both biomarkers, Procoag-PPL® and ETP are commercially available, easy to perform, and do not require a specialized laboratory infrastructure. The Procoag-PPL^®^ clotting time is a commercially available, user-friendly, fully automated, quick, and reproducible technique, which can be installed in any blood coagulation analyzer^33^. The measurement of ETP performed in PPP with the TF 5 pM PPP-Reagent^®^ using the Calibrated Automated Thrombogram-Thrombinoscope® assay is an automated, standardized technique, available in the market worldwide. Important steps toward the standardization of the external quality control procedure have been accomplished^34,35^. The new version of the Calibrated Automated Thrombogram-Thrombinoscope® analyzer, recently presented by the manufacturer, will render this method accessible to hematological laboratories, which are not highly specialized in blood coagulation exploration. A financial analysis remains to be performed so that the benefits of the new score will not be restricted by the cost of the laboratory assessment.

The concept that the assessment of hypercoagulability biomarkers could improve the accuracy of clinical RAMs to stratify ambulatory cancer patients at VTE risk has been tested in previous studies^36^. The original Khonara score for chemotherapy related VTE risk in patients with solid tumors had a sensitivity of 40% and a specificity of 88% in the derivation cohort and a sensitivity of 35.77% and specificity of 89.6% in the validation cohort^37^. The Vienna prediction score for patients with various solid and hematologic malignancies improved the performance of the Khorana RAM by incorporating soluble P-selectin and D-Dimers^26,38,39^. The sensitivity and the specificity of the COMPASS–CAT RAM for CAT in patients with solid tumors were 88% and 52%, respectively^40^. The ROADMAP-CAT lung adenocarcinoma study showed that the introduction of the Procoag-PPL® and MRI of the Calibrated Automated Thrombogram-Thrombinoscope® into the COMPASS-CAT score significantly improved its sensitivity, specificity, and positive predictive value^36^.

Following this line of evidence the next step of the ROADMAP-MM-CAT project is the incorporation of the ROADMAP-MM score into the clinical RAM for more accurate stratification of NDMM patients and administration of thromboprophylaxis in those classified as high-risk of VTE.

The present study has some limitations. The monocentric design did not allow the evaluation of the potential influence of other therapeutic practices or supportive treatments on the predictive capacity of the studied biomarkers. Indeed, 32% of patients received IMiD-based therapy. This might not be representative for countries where triplet therapy (VRd- bortezomib, lenalidomide, dexamethasone) is standard of care. Nevertheless, we should note that biomarkers were assessed at inclusion, before any treatment administration. As a consequence, the design of the ROADMAP-MM-CAT study did not aim to capture the impact of anti-myeloma treatment on cellular and plasma hypercoagulability.

Interestingly, the rate of VTE was not significantly different between the subgroups of patients who received IMid-based treatment as compared to those who received conventional or bortezomib-based treatment. Since patients on IMid-based treatment received the recommended thromboprophylaxis, we assume that the residual VTE risk stems from the limitation of the actual method for the evaluation of VTE risk and suboptimal thromboprophylaxis administration. This hypothesis is being controlled in the ongoing ROADMAP-CAT-MM project. The size of the derivation cohort and the number of VTE events provide sufficient statistical power for the derivation of the new score. However, the sample size did not allow any internal validation of the model and this is the aim of the ongoing ROADMAP-CAT-MM.

The findings of the present study are restricted to the assessment of procoagulant phospholipids and thrombin generation in plasma using only the assays and the reagents employed. The performance of other methods, which measure thrombin generation (i.e., in-house assays, other combinations of reagents, or other techniques available in the market) to evaluate VTE risk in NDMM patients, should be assessed in suitably designed prospective studies. Caution is advised before extrapolating the results of the present study into unselected patient populations with MM.

In conclusion, the prospective ROADMAP-CAT-MM study demonstrates the presence of pronounced cellular hypercoagulability in newly diagnosed chemotherapy naïve patients with symptomatic multiple myeloma, characterized by decreased Procoag-PPL® clotting time, enhanced endothelial cell activation, and exhausted thrombin generation. Among a large number of biomarkers of hypercoagulability, the Procoag-PPL clotting time and the ETP of thrombin generation were found to be independently associated with the risk of VTE and formulated a new score that accurately stratifies patients to high- and intermediate/low-level of VTE risk. The evaluation of these biomarkers is feasible in most hospitals and should be taken into consideration when designing phase III clinical trials that evaluate the efficacy and safety of pharmacological thromboprophylaxis in outpatients with multiple myeloma.
